# Supplementary material for: Dynamic right ventricular and atrial volume responses to exercise in endurance-trained and untrained healthy individuals
Source: PLoS One. 2026 May 20;21(5):e0347745. doi: 10.1371/journal.pone.0347745 (PMC13189338; doi:10.1371/journal.pone.0347745)
Supplement: Attachment — Submitted filename: Response to Reviewers_260218.docx [file pone.0347745.s003.docx]

Dear editor, associate professor Neftali Eduardo Antonio-Villa,

We are grateful for the opportunity to revise, improve, and resubmit our manuscript initially titled “**Right ventricular and atrial volumes decrease in athletes but not in healthy controls during exercise**” (Manuscript No PONE-D-25-57809).

We thank the reviewers for their helpful and constructive comments. We have addressed all the points and changed the manuscript accordingly. The revised manuscript includes a marked-up and an unmarked version. All amendments are below, with additions marked in italics and deletions marked with strike-through.

We sincerely hope that you will find our manuscript suitable for publication in PLOS ONE.

Looking forward to your response at your best convenience.

Yours sincerely,

Björn Östenson, M.D., PhD, First Author

Katarina Steding-Ehrenborg, Associate Professor, PhD, Last Author

**Editor Comment 1 (EC1):** Please ensure that your manuscript meets PLOS ONE's style requirements, including those for file naming.

**Response to EC1:** Thank you for pointing out PLOS ONE’s style requirements. The revised manuscript and all associated files have been prepared in accordance with PLOS ONE’s style requirements, including those for file naming.

**EC2:** We note that you have indicated that there are restrictions to data sharing for this study. For studies involving human research participant data or other sensitive data, we encourage authors to share de-identified or anonymized data. However, when data cannot be publicly shared for ethical reasons, we allow authors to make their data sets available upon request.

**EC3:** In the online submission form, you indicated that the datasets generated and analysed during the current study are not publicly available due to the small sample size and the risk of identifying individual participants but are available from the corresponding author on reasonable request. Data are available from the Cardiac MR Group in Lund, Lund University, Sweden for researchers who meet the criteria for access to confidential data and after additional consent from the research participants. For data access requests, please contact the Cardiac MR Group in Lund via email: cmr-lund@med.lu.se.

All PLOS journals now require all data underlying the findings described in their manuscript to be freely available to other researchers, either 1. In a public repository, 2. Within the manuscript itself, or 3. Uploaded as supplementary information. This policy applies to all data except where public deposition would breach compliance with the protocol approved by your research ethics board. If your data cannot be made publicly available for ethical or legal reasons (e.g., public availability would compromise patient privacy), please explain your reasons on resubmission and your exemption request will be escalated for approval.

**Response to EC2 and EC3:** In accordance with PLOS ONE policy, the dataset underlying the results is now provided as two supporting tables (S1 and S2 Tables) accompanying the revised manuscript.

The supporting tables contain anonymized and complete data necessary to reproduce the analyses and results reported in the study, and its public availability does not breach the protocol approved by the regional ethics committee.

We have updated the Data Availability Statement in the submission system accordingly:

**Updated Data Availability Statement:**

“*All relevant data are within the manuscript and its Supporting Information files.*”

**EC4:** We note that the grant information you provided in the ‘Funding Information’ and ‘Financial Disclosure’ sections do not match.

**Response to EC4:** Thank you for identifying any discrepancies between “Funding Information” and “Financial Disclosure” sections. Complying with PLOS ONE’s submission guidelines, grant information can now only be found in the “Funding Information” section in the submission system, not in the manuscript file.

**EC5:** We note that Figure(s) 1, 2, 3, in your submission contain copyrighted images. All PLOS content is published under the Creative Commons Attribution License (CC BY 4.0), which means that the manuscript, images, and Supporting Information files will be freely available online, and any third party is permitted to access, download, copy, distribute, and use these materials in any way, even commercially, with proper attribution.

**Response to EC5:** We would like to clarify that the figures in question comply with PLOS ONE’s Creative Commons Attribution (CC BY 4.0) licensing requirements:

Figure 1 is an original photograph taken by a co-author (KSE) of the manuscript, who has granted permission for its publication under the CC BY 4.0 license.

Figure 2 is an original figure created by the authors specifically for this manuscript and is also permitted to be published under the CC BY 4.0 license.

Figure 3 is reproduced from a previously published scientific article that is licensed under the CC BY 4.0 license. In accordance with the terms of this license, the figure may be reused, adapted, and redistributed, including for commercial purposes, provided appropriate attribution is given. Full and proper attribution to the original source is included in the figure caption which has been updated to comply with PLOS ONE’s submission guidelines.

We therefore confirm that all figures in the manuscript are compatible with PLOS ONE’s open-access and licensing policies.

**Revised text:**

Page 7, lines 121 – 125:

“**Fig~~ure~~ 3. Respiratory sorting algorithm.** End-diastolic and end-systolic short-axis image stacks at end expiration were generated from real-time CMR images according to the flowchart. Adapted from ~~Edlund et al. in Scientific Reports [13]~~ *Edlund J, Haris K, Ostenfeld E, Carlsson M, Heiberg E, Johansson S, et al. Validation and quantification of left ventricular function during exercise and free breathing from real-time cardiac magnetic resonance images. Sci Rep. 2022;12: 5611. doi:10.1038/s41598-022-09366-8*, licensed under CC BY 4.0; https://creativecommons.org/licenses/by/4.0/.”

**Reviewer #1 Comment 1 (R1C1):** The population appears well controlled for age and sex distribution which is a strength. The authors might consider a description of groups as endurance trained versus untrained healthy individuals; the information provided does not describe competition level, years of training, or a distinction for whether the athletes are recreationally competitive versus elite.

**Response to R1C1:** We have revised the terminology used to describe the study groups in response to the comment. The groups previously referred to as “Athletes” and “Controls” are now consistently described as “Endurance-trained” and “Untrained,” respectively, to more accurately reflect their training status.

In addition, we have expanded the description of the endurance-trained group in the Materials and methods section to include information on competition level preceding study inclusion. While data on years of training unfortunately is not available and therefore cannot be included, data on average weekly training volume during the six months preceding the study inclusion can be found in Table 1.

**Revised text:**

Page 5, lines 72 – 74:

“~~Athletes~~ *ET* were competing in triathlons *at club level with training* *spread across the three training* *disciplines* ~~and endurance training modalities were thus~~ of swimming, cycling, and running.”

**R1C2:** Performance of CMR with exercise obviously requires selected expertise, which is also a strength of this study.

**Response to R1C2:** We thank the reviewer for this positive comment and for recognizing the technical expertise required for performing exercise CMR.

**R1C3:** Strictly speaking, the concept of cardiac chamber remodeling should encompass more than a measure of volumes at end diastole and end systole, and might include more information about chamber mass, or ratio of chamber morphology to cavity dimensions. I would consider removing the comment in the abstract conclusion, and perhaps elsewhere that the study provides understanding of RV remodeling.

**Response to R1C3:** We agree that cardiac chamber remodeling encompasses structural features beyond end-diastolic and end-systolic volumes, including myocardial mass and relationships between wall thickness and cavity dimensions, which were not assessed in the present study.

In response, we have revised the manuscript to temper statements regarding cardiac remodeling. Specifically, references to providing insight into RV remodeling have been removed from the Abstract and revised in the manuscript. We have instead emphasized the dynamic volumetric response of the cardiac chambers during exercise, which more accurately reflects the scope of the study. We also added a clarification to the Limitation section.

These changes have been implemented to ensure that the conclusions drawn are fully supported by the data presented.

**Revised text:**

Page 2, lines 38 – 39:

“This contributes to increased understanding of the RV ~~remodelling~~ *volume adaptations* in ~~athletes~~ *ET*.”

Page 4, lines 44 – 46:

“This exercise-induced cardiac ~~remodelling~~ *volume adaptation* of the whole four-chambered heart is associated with improved endurance performance [4–6].”

Page 4, lines 52 – 53:

“Furthermore, there is a need for increased understanding of the exercise-induced *volume* adaptations of the right ventricle (RV).”

Page 4, lines 62 – 64:

“Thus, the aim of this study was to investigate ~~how~~ the *cardiac* four*-chamber* ~~cardiac chambers, LV, RV, LA, and RA,~~ *volumetric* change from rest to exercise in ~~athletes~~ ET and ~~non-exercising controls~~ *UT* *healthy individuals* while controlling for effects of respiration.”

Page 22, lines 366 – 368:

“*A limitation of this study is that cardiac remodeling was assessed using volumetric measures only. Myocardial mass and wall thickness were not evaluated, which limits conclusions regarding structural remodeling beyond dynamic chamber volume responses during exercise.*”

Pages 22 – 23, lines 383 – 385:

“This study shows differences *in volumetric response* between ~~athletes~~ ET and ~~controls~~ UT during exercise when examining the four cardiac chambers simultaneously and when standardizing for respiratory influences.”

**R1C4:** The paper does contribute carefully measured, chamber volumes from a healthy population, for which more information is clearly welcome. The ability to control for respiratory phase was also an interesting strength. I would have found it additionally helpful for my understanding of this information, given the age range and sex distribution, if the volume data could also be presented indexed for body morphology.

**Response to R1C4:** We have added chamber volume data indexed to body surface area (BSA) to account for differences in body morphology in the revised manuscript. Indexed values are now presented alongside absolute volumes in the Results, and the methodology for BSA indexing has been described in the Materials and methods section. These additions are intended to facilitate interpretation of the findings across the age range and sex distribution of the study population.

**Revised text:**

Page 8, lines 135 – 136:

“*Absolute volumes for all four chambers (LV, RV, LA, and RA) were indexed by body surface area calculated from the Mosteller formula.*”

**R1C5:** It was curious that with information from a CPET study, that moderate and vigorous stages were defined by a percentage of the crude formula for max HR. It appears that some subjects had peak VO2 measured after the imaging study. It would also contextualize the findings to also show the %peak VO2 achieved at each stage, to understand if exercise stages were comparable between the 2 groups.

**Response to R1C5:** Peak oxygen uptake (VO₂peak) was assessed during a separate cardiopulmonary exercise test (CPET) performed on a standard upright cycle ergometer. During the exercise CMR protocol, participants exercised on a custom-built supine cycle ergometer within the MRI scanner. In this study, direct measurement of oxygen uptake was not technically feasible in this setting due to the lack of MR-compatible gas analysis equipment at our center.

Consequently, exercise intensity during CMR was defined using target heart rate ranges based on a percentage of age-predicted maximal heart rate, rather than %VO₂peak. While some participants underwent CPET after the imaging session, VO₂ measurements obtained during CPET could not be directly mapped onto the exercise stages performed in the CMR scanner.

We have clarified this distinction between CPET-derived VO₂ measurements and the exercise CMR protocol in the Materials and methods section to avoid confusion and to better contextualize the interpretation of exercise intensity across study participants. We also acknowledge in the Limitation section that VO_2_ measurements during image acquisition would strengthen the study

**Revised text:**

Page 6, lines 97 – 100:

“Moderate and vigorous exercise *intensities were based on individual heart rate (HR) during image acquisition*, correspond~~ed~~*ing* to 60 and 80 percent of age-predicted maximum ~~heart rate (~~HR~~)~~, *respectively, and* defined according to established exercise intensity terminology [17].”

Page 21, lines 345 – 347:

“Unfortunately, *other means to assess exercise intensity like* exercise duration~~, and~~ workload*, and oxygen consumption (VO2)* at each intensity *during CMR* were not collected in the present study and can therefore not be compared between groups.”

**R1C6:** As per point 3, rather than true chamber remodeling, the paper provides more information about dynamic volume alterations that produce ejection based on intensity of exercise in trained and untrained individuals. Considering this, the presentation of data at a single phase of the respiratory cycle seemed incomplete to understand how the volume data contribute to the performance of the heart under exercising conditions.

**R1C6a:** As the authors discuss on page 17, the data as presented suggest that, in both groups, the primary contributor to increases in CO was the change in HR. Although I would agree, the literature conflict somewhat, to see no change in LV SV or LVEF in the control group would not be expected at all based on hemodynamic studies of exercise. The restricted respiratory phase evaluation may then restrict us from seeing the whole picture, and perhaps presenting the inspiratory phase data, or the data from changes in volumes and LV or RVEF may help us contextualize the data better.

**Response to R1C6a:** We thank the reviewer for this conceptually thoughtful comment regarding both the interpretation of dynamic chamber volume changes during exercise and the influence of respiratory phase.

All volumetric measurements in the present study were performed at end expiration to minimize respiratory variability and improve measurement reproducibility during exercise CMR. We agree that this approach does not capture respiratory-dependent variations in cardiac filling and ejection that occur due to inspiration and expiration. Therefore, our findings primarily reflect dynamic volume changes under standardized respiratory conditions, and not complete fluctuations in stroke volume throughout the respiratory cycle. We have clarified this methodological consideration and its implications in the Materials and methods and Limitations sections.

With respect to the observation that increases in cardiac output were predominantly driven by heart rate, particularly in the untrained group, we acknowledge that this differs from some invasive and echocardiographic studies of exercise. Differences in posture, imaging environment, and respiratory phase standardization may partly account for these discrepancies and are now discussed more explicitly in the manuscript.

**Revised text:**

Pages 7, lines 115 – 116

“*Measuring in the same respiratory phase reduces beat-to-beat variability in chamber volumes related to intrathoracic pressure changes and improves reproducibility.*”

Pages 21, lines 353 – 355

“*Cardiac volume measurements were restricted to end-expiration, resulting in respiratory-dependent fluctuations in stroke volume were not captured. Thus, the reported volumes reflect standardized rather than fully dynamic cardiorespiratory interactions during exercise.*”

Page 20, lines 326 – 328:

“Differences in the mechanism of increased CO could at least partially be explained by different methods used in previous studies*, for example invasive measurements and echocardiography,* as well as body position.”

Page 20, lines 331 – 333:

“*Furthermore, chamber volumes were assessed at a fixed respiratory phase (end expiration), which means that the full effect of respiration was not captured, as respiration interacts with ventricular volumes [10].*”

**R1C6b:** As the paper presents data from all 4 chambers, do the authors subscribe to the constant volume hypothesis of heart function? Is there a shift in RV cardiac output and LV cardiac output contributions during exercise? Such information may help us understand if higher blood volume sequestration in the pulmonary circulation contributes to differences between groups.

**Response to R1C6b:** We agree that the four-chamber data presented are relevant to the concept of the constant volume hypothesis and potential redistribution between right- and left-sided cardiac outputs. However, while our findings suggest differences in right ventricular and atrial volume responses between endurance-trained and untrained healthy individuals during exercise, the study was not designed to assess total heart volume or pulmonary blood volume variation. We have therefore added to the discussion and limitation sections to contextualize the findings.

**Revised text:**

**Page 18, lines 282 – 287**

“*The four-chamber volumetric assessment allows the findings to be considered in the context of the constant volume hypothesis of cardiac function, which proposes that total heart volume remains relatively stable [31]. The observed reductions in RV and RA volumes during exercise in the ET group may reflect more efficient redistribution of blood through the pulmonary circulation rather than four-chamber dilation. However, because total heart volume and pulmonary blood volume were not directly quantified, these interpretations remain speculative and should be explored in future studies.*”

Page 21, lines 355 – 358

“*Total heart volume and pulmonary blood volume variations were not measured. Consequently, potential redistribution of blood between the RV and RA to the LV and LA or from the pulmonary circulation during exercise cannot be directly evaluated.*”

**R2C1:** Sample Size and Statistical Considerations: While the study provides valuable insights, the total sample size (N=33) is relatively small. The authors should briefly address the potential limitations regarding statistical power and risk of bias in the Discussion section. It is important to clarify that these findings are exploratory and specific to this cohort, rather than fully extrapolatable to the broader athletic population.

**Response to R2C1:** We thank the reviewer for this comment regarding sample size and statistical considerations.

We have added a brief discussion of sample size–related limitations to the Discussion section, addressing the potential risks of limited statistical power, bias, and generalizability. We now explicitly state that the findings should be considered exploratory and specific to the studied cohort, and should therefore be interpreted with appropriate caution when extrapolating to broader athletic populations.

We also clarify that an a priori sample size calculation was performed and is described in the Materials and methods section, indicating that a sample size of n=10 was sufficient to detect a medium effect size for the primary outcome measures. While this supports the internal validity of the study, we agree that the total sample size remains a limitation for broader generalization.

**Revised text**:

Page 20, lines 335 – 338:

“*The relatively small sample size of this study could affect statistical power and bias, which may limit generalizability. If underpowered, findings should therefore be considered exploratory and specific to the studied cohort. However, a priori sample size calculation indicated n=10 would be sufficient to* *detect a medium effect size for the primary outcome measures.*”

**R2C2:** Title Refinement: To prevent overgeneralization, I strongly recommend revising the title to more accurately reflect the study’s specific parameters. Given the limited sample size (N=33), the current title implies a universal physiological adaptation that may not be representative of athletic populations globally. I suggest delimiting the title by specifying both the precise cohort of athletes studied and the geographical/regional context where the data was collected (e.g., 'Right ventricular and atrial volumes in a specific cohort of [Type] athletes in [City/Country]: An exploratory study'). Incorporating the geographical location is essential, as environmental, genetic, and regional training factors can influence cardiac physiology. This adjustment will ensure that the findings are interpreted within their correct clinical and regional framework.

**Response to R2C1:** We thank the reviewer for this thoughtful suggestion and for highlighting the importance of avoiding overgeneralization in the title.

We have revised the title to more precisely reflect the specific study population and the exploratory nature of the findings by explicitly referring to endurance-trained individuals rather than athletes in general. This change is intended to better delimit the physiological context of the study without implying universal applicability.

We respectfully chose not to specify a geographical or regional location in the title. While we agree that environmental, genetic, and regional training factors can influence cardiac physiology, we believe that geographic labeling at the city or country level may imply population representativeness that is not warranted for a specialized athletic subgroup such as endurance-trained individuals. Instead, we have clarified the cohort characteristics, recruitment context, and limitations regarding generalizability within the manuscript itself.

We believe that the revised title better reflects the scope and limitations of the present study.

**Revised title:**

“~~Right ventricular and atrial volumes decrease in athletes but not in healthy controls during exercise~~ *Dynamic right ventricular and atrial volume responses to exercise in endurance-trained and untrained healthy individuals*”

**R2C3:** Refining Text and Redundancy: The manuscript would benefit from a more concise presentation. There are instances of redundancy where certain points are reiterated throughout the sections. I recommend a minor editorial review to eliminate these repetitions and improve the flow of the text.

**Response to R2C3:** We thank the reviewer for this helpful suggestion.

In response, the manuscript has undergone a minor editorial revision to reduce redundancy and improve clarity and flow, especially in the Results section.

**Revised text**:

Pages 14 – 15, lines 214 – 222 and 233 – 236:

“Resting HR was lower in ~~athletes~~*ET* ~~compared to~~*than* ~~controls~~*UT*, but both groups *reached*~~achieved~~ similar HR when exercising at moderate and vigorous intensity. Thus, ~~athletes~~ET had a larger increase in HR from rest to exercise (Table 2). LVSV was larger in ~~athletes~~ET than ~~controls~~*UT* at ~~rest, moderate and vigorous exercise~~ *all* intensities and was unchanged with exercise in both *groups* ~~athletes and controls during exercise~~. ~~Likewise,~~ RVSV was *likewise* ~~larger~~*higher* in ~~athletes~~*ET, but decreased during vigorous exercise in ET while remaining unchanged in UT* ~~compared to controls at rest, moderate and vigorous exercise. However, although RVSV was unchanged in controls during exercise, athletes had decreased RVSV during vigorous exercise~~. The change in CO with higher exercise intensity was more pronounced in ~~athletes~~ET compared to ~~controls~~*UT*.

~~LAV~~_~~max~~_~~, LAV~~_~~min~~_ *LA volumes* and LAEF were unchanged ~~from rest to vigorous~~ *with exercise* in both groups (Table 2). *In contrast,* RAVmax decreased in ~~athletes~~*ET* ~~and RAV~~_~~min~~_ ~~was unchanged~~, while ~~RAV~~_~~max~~_ ~~and RAV~~_~~min~~_ RA volumes were ~~both~~ unchanged in ~~controls~~*UT* ~~during exercise~~. RAEF decreased during exercise in both groups.”

**R2C4:** References: Please ensure that all scientific claims are supported by their respective citations. A few areas would benefit from updated references (within the last 5 years) to ensure the study is framed within the most current literature in the field.

**Response to R2C4:** We thank the reviewer for this helpful comment.

We have carefully reviewed the manuscript to ensure that all scientific claims are appropriately supported by citations. Relevant references have been updated to include recent literature from the past five years in order to frame the study with the most current literature in the field.

**Revised text:**

Page 17, lines 261 – 262:

“While the volume response of the LV ~~during~~*with* exercise in healthy participants is well studied, the data is scarce for the RV, LA, and RA *[24]*.”

Page 18, lines 291 – 292:

“As chamber volumes were quantified at end expiration, a slight difference between LVSV and RVSV was expected *as LV and RV volumes have been shown to interact with respiration [10].*”

Page 19, lines 300 – 301:

“Other mechanisms explaining decreased RV volumes during exercise are likely the decreased filling time and enhanced contractility *[33]*.”

Page 20, lines 321 – 326:

“While some studies have shown that there is a similar pattern of SV increase during exercise with a plateau at higher intensities for both ~~athletes~~*ET* and ~~controls~~*UT* [~~33,34~~*36,37*], ~~others have shown a different pattern of SV increase during exercise in athletes with a linear SV increase with increased exercise intensity [35,36]~~ *a recent exercise-CMR review indicate that there is a heterogeneity in SV response to exercise across protocols and cohorts [24].*”

**Added references:**

*24. Trankle CR, Canada JM, Jordan JH, Truong U, Hundley WG. Exercise Cardiovascular Magnetic Resonance: A Review. Journal of Magnetic Resonance Imaging. John Wiley and Sons Inc; 2022. pp. 720–754. doi:10.1002/jmri.27580*

*32. Bowman AW, Kovács SJ. Assessment and consequences of the constant-volume attribute of the four-chambered heart. American Journal of Physiology-Heart and Circulatory Physiology. 2003;285: H2027–H2033. doi:10.1152/ajpheart.00249.2003*

*33. Schweitzer R, de Marvao A, Shah M, Inglese P, Kellman P, Berry A, et al. Establishing Cardiac MRI Reference Ranges Stratified by Sex and Age for Cardiovascular Function during Exercise. Radiol Cardiothorac Imaging. 2025;7. doi:10.1148/ryct.240175*

**Removed references:**

~~35. Schairer JR, Stein PD, Keteyian S, Fedel F, Ehrman J, Alam M, et al. Left ventricular response to submaximal exercise in endurance-trained athletes and sedentary adults. 1992;70(9):930–3.~~

~~36. Gledhill N, Cox D, Jamnik R. Endurance athletes’ stroke volume does not plateau: major advantage is diastolic function. 1994 Sep;26(9):1116–21.~~
